# Somatic Psychoeducational Intervention Is Associated with Increased Oxytocin Levels, Improved Autonomic Function, and Reduced Psychological Distress Symptoms in Medical and Social Care Professionals

**DOI:** 10.3390/healthcare13243236

**Published:** 2025-12-10

**Authors:** Lourdes P. Dale, Audrey N. Dana, Hannah Lamont, Parmida Nazarloo, C. Sue Carter, Stephen W. Porges, Steven P. Cuffe, Donnalea Van Vleet Goelz

**Affiliations:** 1 Department of Psychiatry, College of Medicine-Jacksonville, University of Florida, 580 West 8th St., Tower II, 6th Floor, Jacksonville, FL 32209, USA; 2 Neuroendocrine Unit, Department of Medicine, Massachusetts General Hospital, Harvard Medical School, Boston, MA 02114, USA; 3 Traumatic Stress Research Consortium, Kinsey Institute, Indiana University, 420 North Fess Ave, Bloomington, IN 47408, USA; 4 Department of Psychiatry, University of North Carolina at Chapel Hill, 101 Manning Dr #1, Chapel Hill, NC 27514, USA

**Keywords:** oxytocin, autonomic reactivity, mindfulness, psychological distress, physical stress, polyvagal theory

## Abstract

**Background/Objectives**. Medical and social care professionals (MSCPs) are disproportionately affected by chronic occupational stress, placing them at elevated risk for autonomic dysregulation, affective disorders, and burnout. **Method**. This preliminary study includes data obtained from two samples. The data protocols were approved by University of Florida’s IRB (IRB202200233 approved 24 February 2023 and IRB202401217 approved on 2 October 2024) and registered at ClinicalTrials.gov (NCT05766852 and NCT06580119). With a total sample of 39 female MSCPs, we investigated the effects of the novel Somatic Psychoeducational Intervention (SPI), grounded in Polyvagal Theory, on autonomic reactivity, psychological distress symptoms, and salivary oxytocin levels. Participants engaged in a three- to four-week intervention integrating psychoeducation, interoceptive training, breathwork, and somatic movement. A subset was randomly assigned via a random number generator to allow for comparison of the intervention group (*n* = 8) to control group (*n* = 7). **Results**. Repeated measures ANOVA revealed increases in salivary oxytocin (η_p_^2^ = 0.46) and reductions in autonomic reactivity (η_p_^2^ = 0.24) and psychological distress symptoms (η_p_^2^ ranging from 0.24 to 0.47). Benefits were sustained at one-month follow-up. Subset analyses focused on participants receiving the intervention at the same time found that only the participants in the intervention group improved with regard to autonomic reactivity and symptoms of anxiety and depression. Correlational analyses showed that changes in oxytocin and autonomic reactivity were associated with mindfulness improvements, which in turn predicted symptom reduction. **Conclusions**. These preliminary findings support SPI as a potentially scalable intervention with neurophysiological and psychological benefits. However, high attrition and small subgroup sizes necessitate cautious interpretation.

## 1. Introduction

Medical and social care professionals (MSCPs), which include nurses and child protection care workers, address the physical and psychosocial needs of individuals and communities. The chronic stress experienced by these professionals may lead to increased anxiety, depression, and burnout, which has been supported by research focusing on nurses [1]. There are multiple theories addressing the connection between stress and mental health functioning.

Traditional theories utilize a cognitive top-down approach to stress appraisal. For example, the biopsychosocial model of challenge and threat, grounded in Lazarus’s transactional stress model [2,3,4], conceptualizes stressful situations as complex interactions between an individual’s subjective evaluation of situational demands and their assessment of available coping resources. The model proposes a two-stage appraisal process: primary appraisal, during which situations are evaluated as threatening, harmful, or challenging; and secondary appraisal, during which individuals determine whether their available resources can adequately address situational demands. When perceived resources fall short of demands, individuals experience stress and engage coping strategies, whereas when resources are perceived to match or exceed demands, individuals perceive the situation as being a challenge. This cognitive framework accounts for individual differences in stress responses, as the same situation may be appraised as threatening by one person and challenging by another [2,4].

In contrast, Polyvagal Theory focuses on a bottom-up approach by prioritizing the body’s perception of safety over the cognitive appraisal of safety [5]. Polyvagal Theory [6,7] posits that mental health problems result from autonomic dysregulation and reactivity triggered by faulty neuroception, which is a process whereby the nervous system detects threats outside of conscious awareness [8,9]. Polyvagal Theory posits that neuroception can lead to disruptions in autonomic regulation that can result in maladaptive physiological states such as hypervigilance, anxiety, or shutdown, and can interfere with social engagement and emotional regulation [10,11,12]. A key distinction of Polyvagal Theory is its emphasis on social connection as a primary biological mechanism for downregulating defensive states rather than merely one coping strategy among many, as traditional cognitive models suggest [5].

Oxytocin is increasingly recognized as a key modulator of parasympathetic activity, particularly via its influence on cardiac vagal tone. Preclinical studies [13,14] suggest that oxytocin can regulate autonomic function, improve heart rate variability and promote vagally mediated calming and affiliative states. Thus, oxytocin and the autonomic nervous system may be part of an integrated and highly adaptive system that helps to regulate stress-reactivity [15,16] and may impact the development of psychiatric disorders including eating disorders, schizophrenia, and mood disorders [17]. Research has suggested that PTSD symptoms may be associated with reduced oxytocin and that psychiatric symptoms observed in health care providers may relate to increased self-reported autonomic reactivity [18]. However, we are unaware of research examining this unique combination of variables.

The connection between stress and mental health consequences may relate to dysregulation of the hypothalamic oxytocinergic system. The hypothalamic oxytocinergic system plays a central role in managing and coping with stress [19] by interacting with regulatory areas of the brain and influencing the functioning of the autonomic nervous system [20]. Thus, oxytocin may play a central role in managing and coping with stress [19] by interacting with regulatory areas of the brain and influencing the functioning of the autonomic nervous system [20].

In addition, given that chronic stress may lead to dysregulation of both the oxytocinergic and autonomic nervous systems, individuals experiencing chronic stress may benefit from engaging in psychological interventions, such as mindfulness meditations [21]. Although these interventions may work for some individuals, they may not be effective for everyone because chronic stress has both physical and psychological components and healing is most effective if both components are addressed. Interventions that include both psychological and somatic components [19] to support a sense of social safety and reduce threat [5] may be especially effective in activating the oxytocinergic system. For example, unpublished research from our group revealed significant increases in oxytocin and associated reductions in anxiety in about two-thirds of participants who practiced loving-kindness meditation during a week-long silent retreat.

There is a need to develop and apply accessible somatic approaches to chronic stress that, by definition, involve both the body and mind, and address physical (e.g., autonomic dysregulation and the oxytocin system) and psychological (e.g., mindfulness and symptoms of anxiety, depression, PTSD, and burnout) factors likely to be impacted by stress. Dr. Donnalea Van Vleet Goelz addressed this gap by developing the Somatic Psychoeducational Intervention (SPI), which is grounded in polyvagal theory [6,7] and is an integrative intervention that may improve regulation in neuroendocrine and autonomic systems. The SPI builds on previous research [22] by including psychoeducation concerning polyvagal theory and the autonomic nervous system.

The psychoeducational component of the SPI helps individuals to understand how the body responds to chronic stress, and the potential negative mental and medical consequences of stressful experiences. The SPI also includes breathing techniques found to be useful for the treatment of stress, anxiety, PTSD, depression, stress-related illness, and substance abuse [12]. In addition, it includes elements of Tai Chi to encourage tuning in and paying attention to interoceptive cues and movement patterns while simultaneously maintaining focus on the external world. Research suggests that these ancient Chinese techniques may lead to increased heart rate variability [23] and reduced stress, anxiety, depression, and mood disturbances [24]. Further research is needed to determine whether such methods affect oxytocin-autonomic pathways.

The purpose of the present pilot study was to determine whether SPI could provide simple and effective procedures for helping individuals manage challenges in the real world. Because SPI integrates psychoeducation and somatic techniques designed to enhance autonomic regulation and support psychological resilience, this approach could be especially beneficial to medical and social care professionals who regularly experience chronic stress due to the demands of their work. To assess the potential effects of the intervention, we used objective and subjective measures. Specifically, we measured salivary oxytocin levels, and self-reported autonomic reactivity, mindfulness, and psychological distress symptoms (i.e., anxiety, depression, PTSD, physical stress, work exhaustion, and interpersonal disengagement).

We specifically hypothesized that individuals who receive the intervention will report increases in oxytocin, improvements in mindfulness (particularly interoceptive awareness), and reductions in autonomic reactivity and psychological distress symptoms (i.e., anxiety, depression, PTSD, physical stress, work exhaustion, and interpersonal disengagement). We also hypothesized that the benefits observed in the intervention group would remain through the follow-up assessments and that greater improvements would occur in participants who were initially experiencing greater difficulties and consistently applied the skills they learned during the intervention. In addition, we hypothesized that these changes would not be observed in the control group.

Guided by existing literature suggesting a relationship between oxytocin and emotion regulation, stress reactivity, and physiological flexibility [25], we examined the potential associations between changes in salivary oxytocin levels and shifts in autonomic reactivity, mindfulness, and psychological distress symptoms. We hypothesized that participants demonstrating larger increases in oxytocin and greater reductions in autonomic reactivity from Time 1 to Time 2 would report greater increases in mindfulness and more pronounced reductions in psychological distress symptoms over the same period.

## 2. Method

### 2.1. Participants

**Figure 1:**
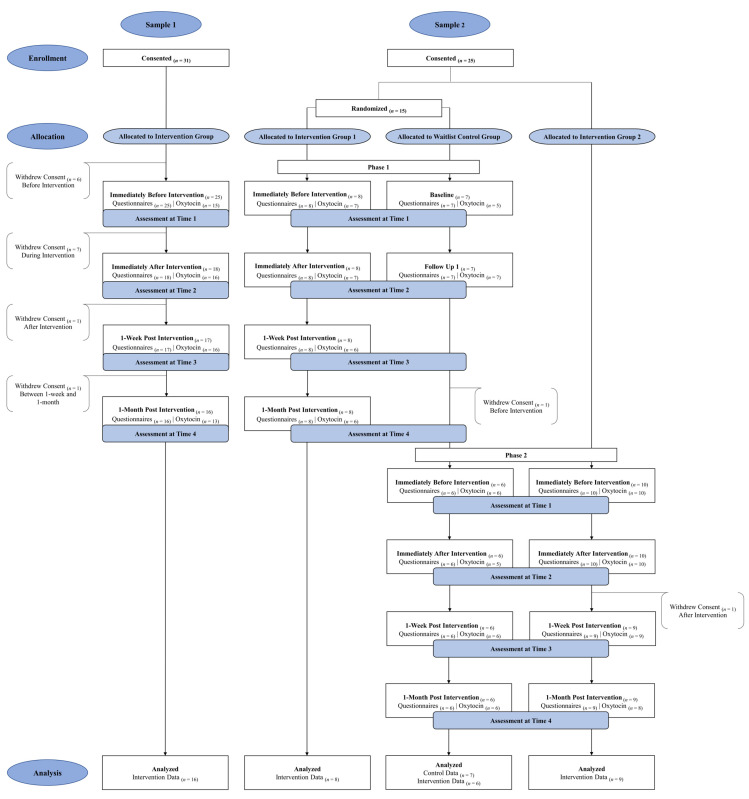
CONSORT Diagram Displaying Participant Flow Through Study.

Figure 1 displays the flow of participants through the study. Below is a detailed explanation of each subsample and the total sample.

#### 2.1.1. Sample 1 (*n* = 16)

Sample 1 included nurses from a major hospital system who completed all classes and evaluations. Although 25 nurses began the intervention, only 16 nurses remained through the final assessments. The nurses reported being female, predominately white (*n* = 12, 80.0%) and varying in age from 24 to 55 years old (*M* = 40.87, *SD* = 10.30). The majority of the nurses (68.8%) reported that their highest level of training was a bachelor’s degree, whereas 12.5% reported an associate’s degree and another 12.5% reported a master’s degree. With respect to annual income, 37.5% of nurses reported earning $60,000 to $80,000; 25.0% reported earning $80,001 to $100,000; 25.0% reported earning $100,001 to $200,000; and 6.3% reported earning over $200,000.

#### 2.1.2. Sample 2 (*n* = 23)

Sample 2 included case workers from a community agency who provide social and community services, including foster care services. The case workers participated in Phase 1 and/or Phase 2 of the study, with Phase 1 preceding Phase 2. During Phase 1, case workers were randomly assigned via a random number generator to one of two groups to evaluate pre- and post-intervention group differences in participants who provided data during this phase. Specifically, participants were not blinded to their group assignment because the participants were aware whether they were currently receiving the intervention (i.e., Intervention Group 1, *n* = 8) or waiting to receive the intervention at a later time (i.e., Waitlist Control Group, *n* = 7). Because one case worker in the waitlist control group dropped from the study after Phase 1, only 6 of the participants from this group received the intervention in Phase 2. Additional recruitment for Phase 2 allowed us to add 10 new participants (i.e., Intervention Group 2), with the enrollment for the Phase 2 intervention group being 16 participants. Of these, 15 participants remained 1-month post-intervention in Phase 2. Thus, 24 participants started the intervention and 23 completed it.

The case workers (ages 25 to 61 years old; *M* = 42.74, *SD* = 9.95) reported being female and primarily identified as white (*n* = 12, 52.2%) or African American (47.8%). The majority (69.6%) reported that their highest degree was a bachelor’s degree, whereas some case workers (26.1%) had a master’s degree. Most case workers (60.9%) reported earning an annual income of less than $60,000, and the remaining reported earning $60,001 to $80,000 (8.7%); $80,001 to 100,000 (17.4%); and $100,001 to $200,000 (13.0%).

#### 2.1.3. Total Sample (*N* = 39)

A total of 48 participants completed the baseline measures (i.e., self-report questionnaire and oxytocin data), but only 39 participants completed all four assessment timepoints. Because of attrition (i.e., 81.3% of baseline participants remained 1-month post intervention), only the data from 39 participants who remained through Time 4 were analyzed in the current study. Additional analyses focused on change from Time 1 to Time 2 for the 6 participants in the control group and compared these participants to the 7 participants who received the intervention at the same time. Although all participants provided salivary samples at every time point, change in oxytocin levels from Time 1 to Time 2 could only be examined for 35 participants and change from Time 1 to Time 4 could only be examined for 29 participants. This discrepancy can be attributed to participant noncompliance (i.e., untestable saliva samples and/or insufficient amount of saliva provided).

### 2.2. Recruitment and Procedures

#### 2.2.1. Sample 1

Data collection occurred from October 2023 to April 2024. Prospective participants in Sample 1 were recruited through a flyer that was distributed and posted throughout the university hospital’s Emergency Department and in-patient units, and it was included in a recruitment email sent by the head of nursing to all the nurses at the hospital. Eligibility criteria included being at least 18 years old and employed as bedside at the hospital. In an effort to increase sample size, participants were provided the option to participate either in-person or virtually. Of the 16 participants in Sample 1, six participants attended in-person, six participants attended virtually, and four participants attended both in-person and virtually. The in-person group classes, which occurred in a conference room at their place of employment, were recorded and later presented to the virtual participants, thus allowing virtual participants to receive the recording of the intervention provided to the in-person participants. Participants, independent of method of participation, attended three 90 min SPI classes (total = 270 min), which occurred weekly over a three-week period. As compensation for participating in this study outside of their work hours, these participants received up to $140 and 4.5 continuing education credits.

#### 2.2.2. Sample 2

Data collection occurred from October 2024 to February 2025. Prospective participants in Sample 2 were recruited through an informational zoom meeting and a follow-up recruitment email with an attached pamphlet that was sent by an administrator to the case workers. Participants were eligible to participate if they were between 18 and 89 years old and were providers at a local agency that provides social and community care services. The group of participants attended one 45 min and three 60 min SPI classes in-person (total = 225 min), which occurred in a conference room at their place of employment during their workday weekly over a four-week period. Because the caseworkers participated for less time and during their work workday, they were provided less compensation than the participants in Sample 1. Specifically, participants in Sample 2 received $85 if they only participated in the intervention, and $120 if they first completed the wait-list control condition and then the intervention.

### 2.3. Somatic Psychoeducational Intervention

Dr. Donnalea Van Vleet Goelz created the SPI, which includes psychoeducation on polyvagal theory, interoceptive training, breathwork, and somatic movement. Thus, this polyvagal-informed intervention should only be provided by therapists with expertise in and the ability to competently integrate the aforementioned components.

Dr. Goelz delivered the intervention to the participants. As part of the SPI, participants were provided education about the nervous system and breathing techniques. Specifically, participants learned about neuroception to increase their awareness of how the nervous system reflexively interprets signals and cues in the environment as safe or threatening [8]. They also learned about interoception to increase their awareness of their bodily reactions when triggered via neuroception and to enhance their ability to emotionally regulate [22,26,27]. Throughout the classes, participants were encouraged to tune into bodily sensations, and to learn how emotional regulation can be accessed by acknowledging their body’s sensations. Regarding breathwork, various Continuum Movement^®^ techniques were introduced, such as the Hu breath, the Cave breath, and the O breath. These breathing techniques are believed to stimulate parasympathetic activity and attenuate sympathetic arousal through engagement of multiple cranial nerves, including the vagus nerve, which is central to autonomic regulation and stress recovery. A subset of participants in Sample 1 and all participants in Sample 2 were taught physical movements focused on exploring, experiencing, and feeling the neural pathways associated with the polyvagal system. They were also taught Tai Chi and fluid movements and were instructed on how to integrate these movements into their daily routines to achieve calming and regulatory benefits. Participants were provided weekly in person reminders to practice the skills learned.

### 2.4. Constructs and Measures

All participants, regardless of group assignment, were assessed at baseline (Time 1) and again immediately following the completion of the SPI (Time 2), which occurred approximately three to four weeks after the initial assessment, depending on the sample. The additional follow-up assessments were provided to the participants receiving the intervention 1-week post SPI (Time 3) and 3–4 weeks post SPI (Time 4). Thus, participants assigned to the waitlist control group completed assessments at Time 1 and Time 2 during their control phase (Phase 1) and completed all four assessments during their intervention phase (Phase 2).

During the baseline assessment, participants self-reported their demographic data (i.e., gender, racial identity, and age). During this assessment and the follow-up assessments (Times 2–4), participants provided saliva samples that assessed peripheral oxytocin levels, and they completed self-report measures assessing their level of autonomic reactivity, mindfulness, and psychological distress symptoms.

#### 2.4.1. Endogenous Salivary Oxytocin

The current study elected to use salivary oxytocin due to its non-invasive method of collection and utility as a potential proxy for central oxytocin [28,29,30]. Salivary OT levels have been used in clinical and behavioral studies to monitor stress reactivity and affiliative processes [30]. The usefulness of measurements of salivary oxytocin levels was previously validated by our group in lactating women [28,31]. Measurements of salivary oxytocin have also been used by other groups to detect acute changes in response to various challenges including sexual activity and exercise [32].

Participants were instructed to refrain from eating, drinking, smoking, or chewing gum for 30–60 min before data collection. Saliva was collected via a passive drool method in which participants were instructed to allow saliva to pool in their mouth and slowly emit it. Sample 1 participants’ saliva was collected using Thermo Scientific™ SpeciMAX™ Saliva Collection Tube (Catalog number A50696) (Thermo Scientific, Waltham, MA, USA). Saliva was collected from Sample 2 participants using the Salimetrics Saliva Collection Aid (Item No. 5016.04) (Salimetrics, State College, PA, USA) and 2 mL Salimetrics Cryovial (Salimetrics Item No. 5004.01). The saliva collection materials are certified for use in low-temperature biobanking and hormone assays.

After collection, saliva samples were immediately stored on ice or in a −18 °C freezer until transferred to a −80 °C freezer for long-term storage. During transfer to long-term storage, saliva samples were stored in an insulated container and kept on ice. The frozen saliva samples from Sample 1 participants were air shipped overnight on dry ice to an out-of-state laboratory for analysis. The frozen saliva samples from Sample 2 were retained and processed at our institution, as the out-of-state laboratory had since physically moved to our facility.

Prior to the assay, the samples were centrifuged (2500× *g* for 20 min at 4 °C), and the supernatant was collected in Eppendorf tubes and stored at −20 °C until analysis. To measure the concentrations of oxytocin (OT), we used a highly sensitive Enzyme Immunoassay kit (ELISA; Arbor Assays Catalog #K048-H1). Per manufacturer documentation [33], the ELISA used has a lower limit detection (LOD) of 16.38 pg/mL for OT, and low cross-reactivity with vasopressin and other neuropeptides. Due to the low oxytocin levels in human saliva, a sample concentration of at least 4-fold was necessary [28]. With this procedure, the oxytocin concentration in saliva fell on a reliable part of the standard curve of the kit [31]. The samples were not extracted as previous research has shown that accurate measurement of OT can be obtained without extraction [28,34,35]. To ensure consistency of assay conditions, each set of participant samples was run concurrently, and all samples were assayed using the same reagents and protocol. All assays were performed by the same researcher, blinded to participant identity and purpose of this study. All oxytocin concentrations included in the final dataset were above the lower limit of detection (16.38 pg/mL), and no values were excluded due to sub-threshold levels.

Saliva samples were analyzed using the Arbor Assays DetectX^®^ Oxytocin Enzyme Immunoassay Kit (Catalog #K048-H1, Lot #OT240316A, Arbor Assays, Ann Arbor, MI, USA). The kit employs a polyclonal antibody specific to oxytocin with minimal cross-reactivity to vasopressin and other neuropeptides. According to manufacturer documentation, the lower limit of detection (LOD) is 16.38 pg/mL, and the assay has been validated for salivary matrix use through internal testing and independent studies [28].

All assays were performed according to the manufacturer’s recommended protocol, with the following additional quality-control steps: Saliva was centrifuged (2500× *g* for 20 min at 4 °C) and supernatants stored at −20 °C prior to analysis. Samples were assayed in duplicate at a 4-fold concentration to ensure values fell within the linear range of the standard curve [28]. Each assay plate included internal controls and all participant samples from the same timepoint batch to minimize inter-assay variability.

All samples were processed by the same researchers using identical reagent lots. Consistent with earlier validations [28,30,31,34], our data yielded salivary OT concentrations above the detection threshold for all participants, supporting assay sensitivity and specificity under non-extracted conditions.

#### 2.4.2. Self-Report Measures

Autonomic reactivity was assessed via the 20-item Body Perception Questionnaire Short Form [36,37,38,39], which is a subjective measure that assesses autonomic symptoms in daily life. The measure asks respondents to indicate via a 5-point Likert scale (1 = *never* to 5 = *always*) the frequency of specific bodily sensations (e.g., *my heart often beats irregularly*; *when I breathe, I feel like I cannot get enough oxygen*; and *after eating I have digestive problems*). Higher scores on this measure, which has been validated with sensor-based systems (e.g., heart rate variability) [38], are indicative of heightened autonomic reactivity [39].

Mindfulness was assessed via the 15-item Five-Facet Mindfulness Questionnaire [40,41]. This measure asks respondents to indicate via a 5-point Likert scale (1 = *never or very rarely true* to *5* = *very often or always true*) how true various statements (e.g., *I’m good at finding words to describe my feelings*; *I notice how foods and drinks affect my thoughts, bodily sensations, and emotions*; and *When I have distressing thoughts or images I am able just to notice them without reacting*) are for them. Higher mean scores reflect higher levels of dispositional mindfulness.

Psychological distress symptoms were measured through well-established measures. Specifically, anxiety symptoms were assessed via the 7-item Generalized Anxiety Disorder—7 [42], which asks respondents to indicate via a 4-point Likert scale (0 = *not at all* to 3 = *nearly every day*) how often they have been impacted in the last 2 weeks by their symptoms (e.g., *Not being able to stop or control worrying*). Depression symptoms were assessed via the 8-item Patient Health Questionnaire—8 [43], which asks respondents to indicate via a 4-point Likert scale (0 = *not at all* to 3 = *nearly every day*) how often in the past 2 weeks they have been impacted by their symptoms (e.g., *Feeling down, depressed, or hopeless*). PTSD symptoms were assessed via the 8-item PTSD Checklist Civilian Version [44], which asks respondents to indicate via a 5-point Likert scale (0 = *not all* to 4 = *extremely*) how impacted they were in the past month by their symptoms (e.g., *Repeated, disturbing, and unwanted memories of the stressful experience*) [44].

Physical stress symptoms were assessed via the unpublished 9-item Physical Stress Symptoms created by two of our authors (available by request). This measure asks respondents to indicate via a 5-point Likert scale (0 = *never* to 4 = *always*) frequency in the last two weeks of the following physical symptoms: *tightness or pressure in head*; *headaches (migraine or tension)*; *teeth grinding*; *jaw tension*, *backaches*; *neck and shoulder pain*; *muscle tightness, cramps, or spasms*; *sweating unrelated to physical activity*; and *clamminess*.

Work exhaustion and interpersonal disengagement were assessed via the 10 items in the corresponding scales of the Professional Fulfillment Index [45]. Respondents were asked to indicate via a 5-point Likert scale (0 = *not at all true* to 4 = *completely true*) the extent to which their job impacted them with respect to work exhaustion (e.g., *a sense of dread when I think about work I have to do*, *physically exhausted at work*, and *emotionally exhausted at work*) and interpersonal disengagement (e.g., *less empathetic with my patients/colleagues*, *less sensitive to others’ feelings/emotions*, and *less connected with my patients/colleagues*) in the last two weeks.

### 2.5. Data Analysis

A priori power analysis via G*Power 3.1.9.7 was used to determine the necessary sample size [46]. For repeated measures ANOVA with a within-between interaction, two groups, and two measurements, it was determined that a sample of 16 is needed to provide 80% power to detect a large-sized effect (Cohen’s f = 0.40) at a significance level of alpha = 0.05 or lower. For two-tailed Pearson’s correlation, a sample size of 29 is needed to provide 80% power to detect a large-sized effect (Pearson’s r = 0.50) at a significance level of alpha = 0.05 or lower.

Responses from all four separate surveys (i.e., Time 1–4) were collected via Qualtrics XM and subsequently exported to IBM SPSS Statistics Version 29.0 [47]. The current study did not employ any corrective techniques to address missing data, which is why we have differing sample sizes depending on the analysis that was run. Descriptive statistics were used to describe the samples with regard to their demographic characteristics (e.g., gender, race, and age) and the range, mean, and standard deviations for the variables. Given the focus on comparing participants to themselves via repeated measures comparisons, the current study elected to use parametric testing techniques. Level of skewness and kurtosis for variables were run to determine whether Pearson’s correlations were the appropriate method of analysis, or if Spearman’s rank correlations would be more appropriate. We elected to use the suggested cutoff values of ≤2 for skewness and ≤7 for kurtosis [48], which has been used in other studies focused on oxytocin [49].

To determine if there were significant improvements in the outcome variables, a series of repeated measures ANOVA were run that looked for statistically significant differences and medium or large effects, as represented by partial eta square (η_p_^2^). First, analyses focused on changes from Time 1 through Time 4 for all the participants who received the intervention and completed all the assessments (*N* = 39). Post hoc pairwise comparison analyses examined which timepoints experienced significant changes for each variable.

Because we hypothesized that the greatest improvements would occur between Time 1 and Time 2 (i.e., pre- to immediately post-intervention), we focused only on these time points for the subsequent analyses. First, ANOVAs determined whether the two groups differed in their response to the intervention as determined by change scores (i.e., Time 2 minus Time 1). Next, Pearson two-tailed correlation analyses explored whether greater improvements (from Time 1 to Time 2) would be found for participants who were struggling the most prior to the intervention (Time 1) and practiced more frequently what they learned during the intervention (between Time 1 and Time 2).

Correlations were also used to examine potential associations between changes from Time 1 to Time 2 in salivary oxytocin levels and shifts in autonomic reactivity, mindfulness, and psychological distress symptoms. Specifically, two-tailed correlational analyses determined whether the participants who exhibited greater increases in oxytocin and decreases in autonomic reactivity from Time 1 to Time 2 also reported greater increases in mindfulness and decreases in psychological distress symptoms from Time 1 to Time 2. Post hoc analyses explored whether participants who improved from Time 1 to Time 2 differed from those who did not improve.

To examine the efficacy of the intervention, additional analyses focused on Sample 2 participants from the same period (i.e., phase 1) to determine whether those that received the intervention differed from those in the control group with regard to their baseline functioning and change in functioning from time 1 to time 2. First, one-way ANOVA explored whether the intervention and control group exhibited baseline differences with respect to their oxytocin levels or any of the psychological distress symptoms. Second, repeated measures ANOVA explored possible interaction effects suggesting group differences between the control group (*n* = 7) and the intervention group (*n* = 8) from Time 1 to Time 2. We were only able to examine these differences in self-report measures, as we lacked the statistical power to examine group differences in oxytocin levels because of the limited number of participants with complete data (*n* = 11).

## 3. Results

SPI was well received by the participants who received the intervention (*N* = 39). Additionally, no adverse or unintended effects were observed or reported during the intervention sessions or data collection assessments.

### 3.1. Analyses Focused on Change from Time 1 to Time 4 for the Total Sample

**Figure 2:**
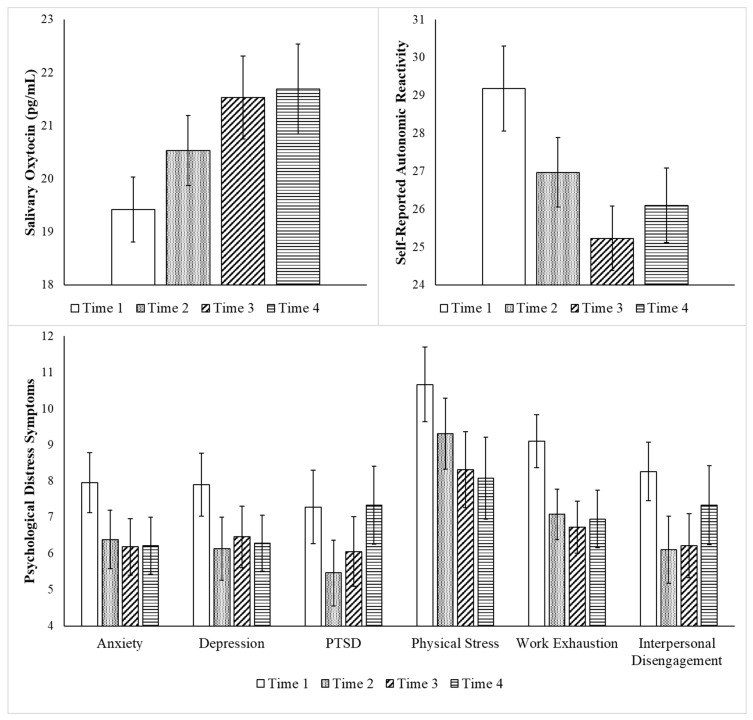
Benefits of the Intervention: Change from Time 1 through Time 4. Legend. Repeated measures ANOVA focused on change from Time 1 through Time 4 for all the participants who had data for all four timepoints (*N* = 39, except for oxytocin *n* = 29). Time 1 was assessed pre-intervention, Time 2 was assessed immediately post-intervention, Time 3 was assessed 1-week post-intervention, and Time 4 was assessed 3–4 weeks post-intervention. Statistically significant improvements were found with regard to all variables, except for mindfulness. Bar graph displays mean values and error bars represent standard error of mean.

Table 1 demonstrates that the participants varied with regard to their baseline (Time 1) functioning. As reported in Table 2 and Figure 2, repeated measures ANOVA assessing change focused on 39 participants who had complete data from Time 1 through Time 4 found improvements in all variables, except mindfulness, as indicated by the medium and large effect sizes. As reported in Table 2, repeated measures analyses focused on the 29 participants (i.e., 12 nurses and 17 case workers) with oxytocin data across all four timepoints showed statistically significant increases in oxytocin from Time 1 through Time 4. Post hoc comparisons indicated that statistically significant increases in oxytocin occurred from baseline to each post-intervention timepoint (Times 2–4).

Although statistically significant increases in mindfulness were not found, post hoc repeated measures ANOVA indicated that there were statistically significant increases in one aspect of mindfulness, the observing of inner events, *F*(3, 36) = 3.13, *p* = 0.037; η_p_^2^ = 0.21. There was a statistically significant improvement in the observation of inner events between Time 1 and Time 2, that was no longer detected at Times 3 and 4.

Consistent reductions in autonomic reactivity and symptoms of depression, physical stress, and work exhaustion were found from Time 1 through Time 4. PTSD and interpersonal disengagement symptoms decreased through Time 3 and then increased at Time 4, whereas anxiety symptoms did not statistically decrease until Time 3.

### 3.2. Analyses Focused on Change from Time 1 to Time 2

Table 1 also reports the skewness and kurtosis of the study variables, which supports the use of Pearson two-tailed correlational analyses focused on change from Time 1 to Time 2. First, the ANOVA analyses focused on understanding whether the two groups differed in their response to the intervention found that the only group difference was with regard to change in oxytocin. As evident from the mean scores presented in Table 3, the participants in Sample 1 exhibited an increase in oxytocin that was not found in the participants in Sample 2.

Pearson two-tailed correlational examined whether greater improvements would be found for participants who were struggling the most prior to the intervention (Time 1) or practiced more frequently what they learned during the intervention. Given the group differences, analyses related to change in oxytocin only focused on the 15 participants in Sample 1 with data from Time 1 to Time 2. The results of these Pearson correlation analyses found that change in oxytocin levels was not related to their baseline levels (*r* = −0.27, *p* = 0.335), but was related to how often they practiced (*r* = 0.55, *p* = 0.033), as participants who practiced more frequently exhibited greater increases.

Further analyses focused on the total sample found statistically significant correlations suggesting that participants struggling the most at Time 1 reported greater improvements in autonomic reactivity (*r* = −0.58, *p* < 0.001), mindfulness (*r* = −0.43, *p* = 0.003), anxiety (*r* = −0.55, *p* < 0.001), depression (*r* = −0.34, *p* = 0.017), PTSD (*r* = −0.53, *p* < 0.001), physical stress (*r* = −0.38, *p* = 0.009), and work exhaustion (*r* = −0.35, *p* = 0.014), but not in interpersonal disengagement (*r* = −0.19, *p* = 0.120). The results also indicated that participants who practiced more frequently reported greater improvements, including increased mindfulness (*r* = 0.38, *p* = 0.012) and reduced autonomic reactivity (*r* = −0.38, *p* = 0.012) and depression symptoms (*r* = −0.35, *p* = 0.018).

Additional analyses focused on determining whether participants demonstrating greater reductions in autonomic reactivity from Time 1 to Time 2 would report greater increases in mindfulness and more pronounced reductions in psychological distress symptoms over the same period. Analyses focused on Sample 1 did not find a statistically significant association between change in oxytocin and change in autonomic reactivity (*r* = −0.37, *p* = 0.169) but it did suggest trend whereby increases in oxytocin were related to increased mindfulness (*r* = 0.48, *p* = 0.070). However, analyses focused on the total sample found that reductions in autonomic reactivity was significantly related to increased mindfulness (*r* = −0.36, *p* = 0.012), which was found to be associated with decreased symptoms of anxiety (*r* = −0.35, *p* = 0.014), depression (*r* = −0.31, *p* = 0.028), and PTSD (*r* = −0.33, *p* = 0.020).

**Figure 3:**
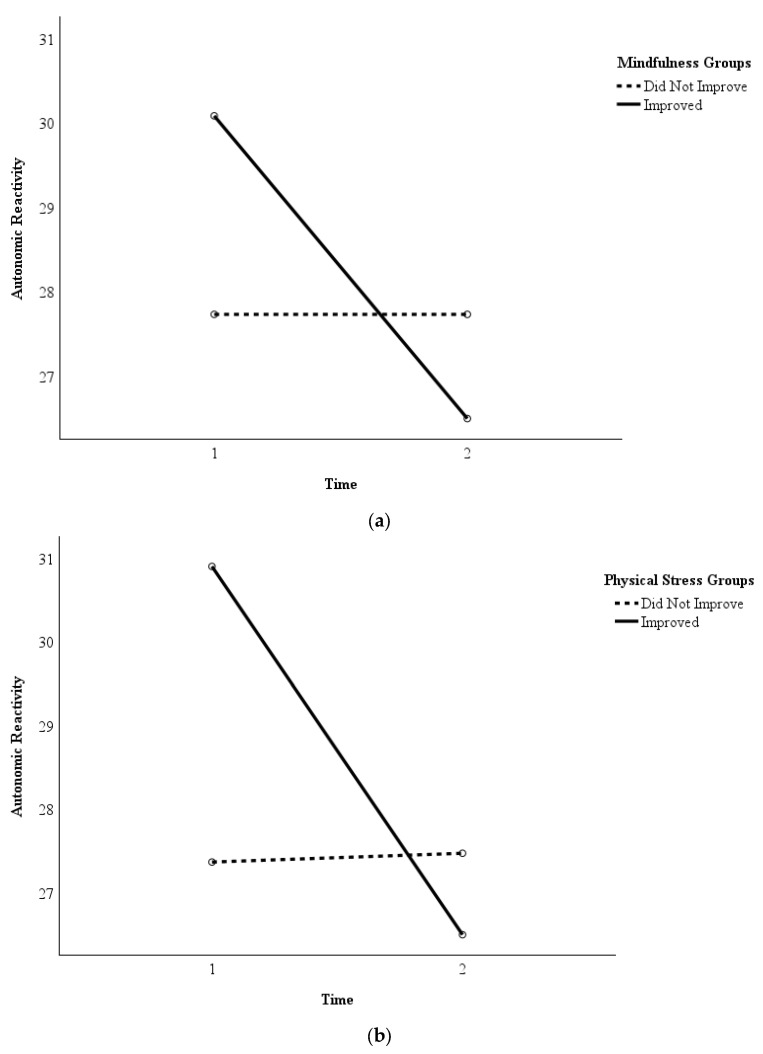
(**a**) Autonomic Reactivity in Participants Who Improved in Mindfulness versus Those Who Did Not. Legend. Repeated measures ANOVA determined whether the participants in the group that improved (*n* = 24) in mindfulness reported greater decreases in their autonomic reactivity from Time 1 (pre-intervention) to Time 2 (immediately post-intervention) than the participants in the group who did not improve (*n* = 15). The group that reported improvements in mindfulness reported greater decreases in autonomic reactivity than the group that did not report improvements. (**b**) Autonomic Reactivity in Participants Who Improved in Physical Stress Symptoms versus Those Who Did Not. Legend. Repeated measures ANOVA determined whether the participants in the group that improved (*n* = 20) in physical stress symptoms reported greater decreases in their autonomic reactivity from Time 1 (pre-intervention) to Time 2 (immediately post-intervention) than the participants in the group who did not improve (*n* = 19). The group that reported improvements in physical stress symptoms reported greater decreases in autonomic reactivity than the group that did not report improvements.

In addition, analyses indicated that decreased autonomic reactivity was associated with reduced symptoms of depression (*r* = 0.48, *p* = 0.001), PTSD (*r* = 0.49, *p* < 0.001), physical stress (*r* = 0.51, *p* < 0.001), and work exhaustion (*r* = 0.44, *p* = 0.002). Post hoc repeated measures ANOVA found statistically significant differences in autonomic reactivity between the participants who did and did not improve from Time 1 to Time 2 with regard to mindfulness (*F* = 5.39, *p* = 0.026, η_p_^2^ = 0.13) and physical stress (*F* = 9.96, *p* = 0.003, η_p_^2^ = 0.21). Figure 3a,b depict this pattern, as participants who improved in mindfulness (*n* = 24) and physical stress (*n* = 20) reported greater decreases in their autonomic reactivity from Time 1 to Time 2 than the participants who did not improve (*n* = 15 and *n* = 19, respectively). The same pattern was observed to be approaching statistical significance regarding measures of PTSD (improved *n* = 22, did not improve *n* = 17; *F* = 3.15, *p* = 0.084, η_p_^2^ = 0.08) and work exhaustion (improved *n* = 26, did not improve *n* = 13; *F* = 3.57, *p* = 0.067, η_p_^2^ = 0.09).

### 3.3. Analyses Comparing Control and Intervention Subgroups from Time 1 to Time 2

**Figure 4:**
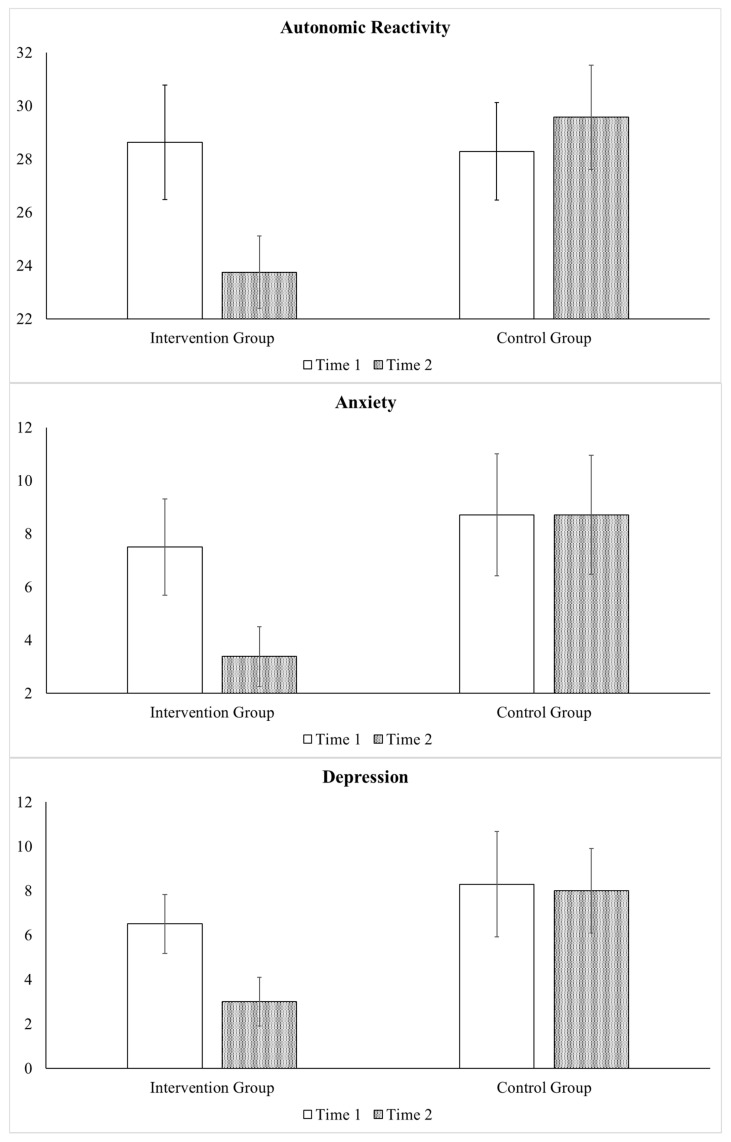
Benefits of the Intervention: Comparison of Intervention and Control Group. Legend. Repeated measures ANOVA compared the participants in the control group (*n* = 7) to the participants who received the SPI during the same time period (*n* = 8) from Time 1 (pre-intervention) to Time 2 (immediately post-intervention). Improvements were only found in the intervention group with respect to autonomic reactivity and symptoms of anxiety and depression. Bar graph displays mean values and error bars represent standard error of mean.

One-way ANOVA determining whether there were baseline differences between the participants in the control group (*n* = 7) and the participants who received the intervention (*n* = 8) during the same time period (phase 1) found that the groups did not differ with regard to oxytocin levels or any of the psychological distress symptoms. Repeated measures ANOVA focused on comparing the 7 participants in the control group to the 8 participants in the intervention group from Time 1 to Time 2. Large interaction effects were found regarding autonomic reactivity (*F* = 9.38, *p* = 0.009; η_p_^2^ = 0.42), anxiety (*F* = 6.22, *p* = 0.027; η_p_^2^ = 0.32), and depression (*F* = 5.74, *p* = 0.009; η_p_^2^ = 0.31). Specifically, as displayed in Figure 4, improvements were only found in the intervention group, as they exhibited greater decreases in autonomic reactivity (Intervention Group mean difference = −4.88 and Control Group mean difference = 1.28), and symptoms of anxiety (Intervention Group mean difference = −4.12 and Control Group mean difference = 0.00) and depression (Intervention Group mean difference = −3.50 and Control Group mean difference = −0.29). Of note, a similar pattern also occurred with regard to other variables, even though they were not statistically significant due to small sample sizes.

## 4. Discussion

Medical and social care professionals (MSCPs) may experience high rates of anxiety, depression, and burnout [1], which can affect their patient care and ability to remain in their current positions. They also may experience higher levels of autonomic reactivity, which we have previously described in medical doctors [18]. Because oxytocin is believed to modulate stress reactivity and facilitate restoration following periods of challenge [19,50], we investigated whether oxytocin levels would be increased and self-reported autonomic reactivity would be decreased following the Somatic Psychoeducation Intervention (SPI) designed to increase awareness of how stress negatively impacts autonomic and emotional regulation and also teach participants strategies for improving these difficulties. We also investigated whether the MSC professionals would report increased mindfulness and decreased psychological distress symptoms (i.e., anxiety, depression, PTSD, physical stress, work exhaustion, and interpersonal disengagement).

Our preliminary findings suggest that the SPI was well received by the MSC professionals, as they reported enjoying the classes and using what they learned in their personal and professional lives with positive effects. In a smaller subgroup, we found that those that received the SPI reported greater reductions in autonomic reactivity and symptoms of anxiety and depression than those in the control group. When the SPI was tested with the larger sample, we found that the intervention was associated with consistent increases in oxytocin levels across the four time points, including samples taken after the termination of formal training. However, group comparison found that the participants in Sample 1 exhibited an increase in oxytocin that was not found in the participants in Sample 2. We suspect that these differences may relate to two external factors. First, the individuals in Sample 1 participated outside of their work hours and had the flexibility to choose the sessions that worked best for them. Conversely, the individuals in Sample 2 participated at a pre-determined time in the middle of their workday, and thus often discussed work-related stressors, including upcoming deadlines and traumatic experiences during field work. For example, many of these individuals reported being very impacted and disturbed by recent changes in the political environment and policies that they perceived as detrimental to their clients. Thus, these experiences may have negatively impacted their ability to feel calm and safe, as suggested by the lack of significant change in their oxytocin levels.

In addition, our results suggested that the intervention was associated with other consistent improvements, as participants reported statistically significant reductions in autonomic reactivity and psychological distress symptoms (i.e., anxiety, depression, PTSD, physical stress, work exhaustion, and interpersonal disengagement). Although preliminary, our findings are consistent with prior research [51] suggesting that somatic interventions may reduce PTSD symptoms among individuals diagnosed with PTSD.

Our results further indicated that participants who were struggling the most at baseline reported greater increases in mindfulness and decreases in autonomic reactivity and psychological distress symptoms (i.e., anxiety, depression, PTSD, physical stress, and work exhaustion). In addition, the participants who practiced what they had learned more frequently reported exhibited greater increases in oxytocin and improvements in autonomic reactivity, mindfulness, and depression symptomatology. Although we did not find statistically significant improvements in mindfulness, we did find significant improvements in observation of inner events, which is the facet of mindfulness that is directly addressed during the SPI via its focus on interoception. Thus, our combined results support the efficacy of SPI, especially for individuals struggling the most and/or those who practice what they learned.

Although prior research [50] suggests that oxytocin and autonomic reactivity may function together as part of an integrated stress-regulation system, our study could not support this finding. This was because the analyses focused on change in oxytocin levels included only Sample 1 participants and thus lacked statistical power to find significant effects. Although not statistically significant, we found moderate strength correlation between the change in oxytocin and autonomic reactivity (*r* = −0.37). Thus, further research is necessary to determine if these systems are related or operate along independent pathways that may or may not be associated with similar improvements.

We found that increases in OT and decreases in autonomic reactivity, which may reflect modulation of the OT-CRF axis, were associated with improvements in mindfulness. This pattern is consistent with the proposed model that SPI may facilitate oxytocinergic engagement [52] which may support enhanced interoceptive awareness and regulatory capacity. The shifts in mindfulness may help explain downstream observed benefits in psychological outcomes, such as reduced anxiety, depression, and PTSD symptoms. These preliminary findings are consistent with prior research [16,50] suggesting that interventions addressing aspects of mindfulness, such as those integrated in the SPI, may increase oxytocin levels and facilitate neuroplasticity in brain regions involved in emotion regulation.

The improvements in autonomic reactivity were expected given that SPI targets autonomic flexibility and resilience via somatic movement and breath-based techniques. Specifically, our intervention’s focus on breath and interoceptive awareness may have amplified vagal engagement by stimulating vagal afferents and aligning with OT’s anxiolytic and anti-inflammatory properties [19]. We found that decreases in self-reported autonomic reactivity were directly associated with decreases in depression, PTSD, physical stress, and work exhaustion symptomatology. Thus, our results are consistent with research and theory [7,52] emphasizing the role of vagal regulation in managing bodily states, which in turn may impact emotion regulation. Our findings also contribute to the existing research suggesting that interventions targeting autonomic regulation, such as Tai Chi and breathwork, lead to improvements in heart rate variability and reduced sympathetic dominance [12,23], as would be predicted by Polyvagal Theory [7].

Regarding occupational functioning, we found that improvements in work exhaustion were directly related to reduced autonomic reactivity, while improvements in interpersonal disengagement were not correlated with physiological markers. This suggests that the improvement in interpersonal disengagement may be an intervention-specific effect, potentially due to group cohesion or shared experience. This would follow from the results of prior studies suggesting that group-based interventions and co-regulation can bolster prosocial behaviors and mitigate burnout [53].

Taken together, our findings align well with predictions from Polyvagal Theory and the proposed SPI model. However, it is important to acknowledge that alternative theoretical frameworks may also account for these results. The biopsychosocial model of challenge and threat [2,3,4], which emphasizes cognitive appraisal processes, offers a complementary lens through which to interpret our observed improvements in autonomic reactivity and psychological outcomes. According to this framework, the SPI may have facilitated a shift in participants’ appraisal patterns, enabling them to perceive occupational stressors as more manageable challenges rather than overwhelming threats. This reappraisal process could explain the downstream reductions in anxiety, depression, and PTSD symptoms through cognitive–emotional pathways rather than solely through bottom-up autonomic regulation. Similarly, the improvements in mindfulness and oxytocin levels observed in our study may reflect top-down cognitive regulation processes, wherein enhanced awareness and reappraisal of internal states influence both neuroendocrine and autonomic functioning [2,3].

The relationship between reduced autonomic reactivity and decreased work exhaustion could also be understood through this cognitive appraisal lens, as individuals who perceive their resources as adequate to meet workplace demands would be expected to show both reduced physiological stress markers and improved occupational outcomes. While our emphasis on Polyvagal Theory provides a neurophysiologically grounded framework for understanding these mechanisms, the convergence of our findings with predictions from cognitive appraisal models suggests that multiple pathways, both bottom-up autonomic regulation and top-down cognitive processes, may interact to produce the observed therapeutic effects. Future research employing designs that can disentangle these pathways would strengthen our understanding of the mechanisms underlying SPI’s efficacy.

## 5. Limitations

We acknowledge that there are a range of contextual and demographic factors which may have impacted the study’s findings. As previously mentioned, this may have been especially true for the participants in Sample 2 who frequently reported experiencing stress related to their workday and did not exhibit significant increases in oxytocin. Another significant limitation relates to the makeup of the sample, as all participants self-identified as female. This overrepresentation of individuals who identify as female is consistent with population estimates, as it is estimated that most registered nurses and child welfare workers identify as female [54,55]. However, this does not allow us to generalize these findings to individuals identifying as other genders.

Another limitation of our pilot study relates to attrition rates that led to a relatively small final sample size of 39 participants. Despite the initial desire to participate in the intervention, a few participants dropped out of the study. This was a particular problem in nurses who reported difficulty committing their free time to the intervention, even when offered the option of attending classes virtually. In contrast, it was easier to get commitment from the childcare workers, possibly because they were allowed to do the intervention during their workday. This underscores the importance of providing workers in high-stress environments with the opportunity to participate in interventions aimed at improving wellbeing during the workday.

Although several statistically significant results with moderate-to-large effect sizes were observed, the small sample size, especially with regard to the control and intervention groups, limits confidence in these findings. Although most of the analyses are focused on comparing individuals to themselves, the choice to run a parametric test may have led to a type II. Additionally, given the exploratory nature of these analyses and the number of comparisons conducted, the potential for Type I error inflation is possible. Improvements may have been related to other factors beyond the intervention, including the provider’s ability to meaningfully connect with the participants and their stories, and the social support that many of the participants may have experienced while being part of a group. Thus, all findings should be interpreted as preliminary and hypothesis-generating, rather than conclusive evidence of treatment efficacy. To confirm these findings, additional research should be conducted with a sample that is demographically diverse and large enough to appropriately compare individuals who receive the intervention to those in a control group.

Another limitation refers to the oxytocin data, as some salivary samples were contaminated by blood or were too small in volume to allow them to be assayed. This was particularly a problem when comparing the intervention and control groups. However, we were able to conduct repeated measures ANOVA on most oxytocin samples, thus allowing for within subject comparisons. Given the sensitivity of the oxytocin assays, another limitation is that some samples were assayed at different points in time and/or on two different machines, thus limiting the type of statistical analyses that could be conducted. Therefore, the current study primarily focused on within subject differences and only examined group differences with samples that were assayed at the same time.

Our study is also limited by its reliance on self-report measures, as this may have contributed to response bias and/or regression towards the mean [56]. However, the finding that improvements in these self-report measures were associated with increases in oxytocin, which is a biological marker shown to be relevant to behavioral states and stress in many other studies [19], supports the preliminary hypothesis that the SPI has the potential to be beneficial. The addition of objective physiological measures (e.g., heart rate variability) might enhance the robustness of findings in future studies.

## 6. Conclusions

The results of this pilot study suggest that the improvements in the psychological distress symptoms (i.e., anxiety, depression, PTSD, physical stress, work exhaustion, and interpersonal disengagement) may relate to the intervention described here. However, it remains unclear which specific components of the SPI (e.g., Tai Chi, breathwork, and psychoeducation) are responsible for the observed improvements. Given its multimodal nature, future studies are needed to isolate the most effective elements of the SPI.

This study also suggests that the effects of the SPI could be indirect. Specifically, decreased autonomic reactivity was associated with decreases in depression, PTSD, physical stress, and work exhaustion symptoms. In addition, both decreases in autonomic reactivity and increases in oxytocin levels were associated with increased mindfulness, which in turn, was linked to decreases in anxiety, depression, and PTSD symptoms.

The results of this pilot study support the possible value of SPI, an inexpensive and accessible therapy that focuses on increasing bodily awareness through psychoeducation, movement techniques, and relaxation. The study is novel in assessing oxytocin as a biomarker that may relate to changes in behavior and autonomic reactivity, possibly providing clues to the mechanisms through which SPI works. By addressing both the mind and body, SPI may be particularly beneficial in reducing the negative consequences of stress for individuals struggling with psychological distress symptoms that may relate to increased autonomic reactivity. The study also offers preliminary support for the possible usefulness of self-report measures, such as the Body Perception Questionnaire [36,37,38,39], to determine who is most in need and likely to benefit from interventions. Most importantly, the finding that SPI was associated with a decrease in two aspects of burnout (i.e., work exhaustion and interpersonal disengagement) suggests that this intervention may improve the wellbeing of the MSCPs, which may allow them to provide better patient and client care.

## Figures and Tables

**Table 1:** Descriptive Statistics for the Variables.

Variables			Skewness		Kurtosis	
	Range	*M* (*SD*)	Statistic	Standard Error	Statistic	Standard Error
Oxytocin	15.67–33.68	19.95 (4.33)	1.88	0.39	3.24	0.76
Autonomic Reactivity	20–50	29.18 (7.06)	1.02	0.38	1.29	0.74
Mindfulness	2.53–4.60	3.45 (0.49)	0.38	0.38	−0.26	0.74
Psychological Distress Symptoms						
Anxiety	0–19	7.95 (5.35)	0.45	0.38	−0.86	0.74
Depression	1–22	7.90 (5.44)	1.01	0.38	0.51	0.74
PTSD	0–22	7.28 (6.42)	0.77	0.38	−0.78	0.74
Physical stress	0–25	10.67 (6.39)	0.34	0.38	−0.65	0.74
Work Exhaustion	1–16	9.10 (4.58)	0.71	0.38	−1.24	0.74
Interpersonal Disengagement	0–19	8.26 (5.23)	0.15	0.38	−0.44	0.74

*N* = 39, except for oxytocin *n* = 38.

**Table 2:** Benefits of the Intervention: Change from Time 1 through Time 4.

Variables	Time 1 *M* (*SD*)	Time 2 *M* (*SD*)	Time 3 *M* (*SD*)	Time 4 *M* (*SD*)	*F*	*p*	η_p_^2^
Oxytocin	19.42 ^abc^ (3.24)	20.53 ^a^ (3.49)	21.53 ^b^ (4.14)	21.69 ^c^ (4.55)	7.49	<0.001	0.46 ^L^
Autonomic Reactivity	29.18 ^ab^ (7.06)	26.97 ^a^ (5.82)	25.23 ^b^ (5.19)	26.10 (6.10)	3.74	0.020	0.24 ^L^
Mindfulness	51.74 (7.29)	52.80 (6.91)	52.74 (6.32)	53.21 (7.08)	0.83	0.486	0.07 ^M^
Psychological Distress Symptoms							
Anxiety	7.95 ^a^ (5.35)	6.38 (4.98)	6.18 ^a^ (5.03)	6.21 (5.21)	3.96	0.015	0.25 ^L^
Depression	7.90 ^a^ (5.44)	6.13 ^a^ (5.36)	6.46 (5.43)	6.28 (5.00)	3.79	0.018	0.24 ^L^
PTSD	7.28 (6.42)	5.46 (5.58)	6.05 (6.11)	7.33 (7.07)	3.76	0.019	0.24 ^L^
Physical stress	10.67 ^ab^ (6.39	9.31 (6.07)	8.3 ^a^ (6.59)	8.08 ^b^ (7.13)	5.04	0.005	0.30 ^L^
Work Exhaustion	9.10 ^abc^ (4.58)	7.08 ^a^ (4.41)	6.72 ^b^ (4.58)	6.95 ^c^ (4.95)	10.75	<0.001	0.47 ^L^
Interpersonal Disengagement	8.26 ^ab^ (5.23)	6.10 ^a^ (6.04)	6.2 ^b^ (5.68)	7.33 (7.12)	9.47	<0.001	0.44 ^L^

*N* = 39, except for oxytocin *n* = 29. Timepoints with the same superscript lowercase letter (i.e., a,b,c) are significantly different from another; a timepoint may carry multiple letters (e.g., ab) if it differs significantly from multiple other timepoints. η_p_^2^ = Partial eta squared. Medium Effect (indicated by superscript M): η_p_^2^ ≥ 0.06, Large Effect (indicated by superscript L): η_p_^2^ ≥ 0.14. Time 1 was assessed pre-intervention, Time 2 was assessed immediately post-intervention, Time 3 was assessed 1-week post-intervention, and Time 4 was assessed 3–4 weeks post-intervention.

**Table 3:** Comparison of Intervention Response by Sample.

Variables	Sample 1 *M* (*SD*)	Sample 2 *M* (*SD*)	*F*	η^2^
Oxytocin	2.15 (2.28)	0.14 (1.66)	9.17 **	0.22
Autonomic Reactivity	−2.00 (5.48)	−2.35 (4.68)	0.05	0.00
Mindfulness	0.88 (6.50)	1.17 (4.14)	0.03	0.00
Psychological Distress Symptoms				
Anxiety	−1.06 (6.23)	−1.91 (4.21)	0.26	0.01
Depression	−2.31 (3.24)	−1.39 (3.61)	0.67	0.02
PTSD	−2.81 (6.32)	−1.13 (3.15)	1.21	0.03
Physical stress	−1.94 (3.94)	−0.96 (3.78)	0.61	0.02
Work Exhaustion	−2.75 (2.57)	−1.52 (2.61)	2.12	0.05
Interpersonal Disengagement	−3.63 (3.91)	−1.13 (4.15)	3.57	0.09

*N* = 39, except for oxytocin *n* = 35. ** *p* < 0.01. η^2^ = Eta squared. Small Effect: η^2^ ≥ 0.01, Medium Effect: η^2^ ≥ 0.06, Large Effect: η^2^ ≥ 0.14.

## Data Availability

The datasets generated and/or analyzed during the current study are not publicly available to protect the privacy of participants, who could be more easily identified via demographic characteristics in our small sample. Interested researchers may reach out to the corresponding author regarding the availability of the data for reproducing the results.
